# Juvenile/Peripubertal Exposure to Omega-3 and Environmental Enrichment Differentially Affects CORT Secretion and Adulthood Stress Coping, Sociability, and CA3 Glucocorticoid Receptor Expression in Male and Female Rats

**DOI:** 10.3390/nu16142350

**Published:** 2024-07-20

**Authors:** Julie Raymond, Alexandre Morin, Meenakshie Bradley-Garcia, Hélène Plamondon

**Affiliations:** Behavioural Neuroscience Group, School of Psychology, University of Ottawa, 136 Jean-Jacques Lussier, Ottawa, ON K1N 6N5, Canada; jraym082@gmail.com (J.R.); amori092@uottawa.ca (A.M.); mbrad048@uottawa.ca (M.B.-G.)

**Keywords:** fish oil supplementation, juveniles, sex-dependent effects, enriched environment, coping responses, sociability/social recognition, conditioned fear

## Abstract

In adult rats, omega-3 supplementation through fish oil (FO) and environmental enrichment (EE) have shown beneficial effects on cognition and stress regulation. This study assessed sex-specific effects of FO and EE during adolescence, a period critical for brain maturation, on adulthood coping mechanisms, sociability, and glucocorticoid regulation. An amount of 64 Wistar rats [n = 32/sex; postnatal day (PND) 23] were assigned to supplementation of control soybean oil (CSO) or menhaden fish oil (FO; 0.3 mL/100 g) from PND28 to 47 and exposed to EE or regular cage (RC) housing from PND28 to 58, with their blood corticosterone (CORT) levels being assessed weekly. As adults, exposure to repeated forced swim tests (FSTs; PND90–91) enabled analysis of coping responses, while socioemotional and memory responses were evaluated using the OFT, EPM, SIT, and Y maze tests (PND92–94). Immunohistochemistry determined hippocampal CA1/CA3 glucocorticoid receptor (GR) expression (PND95). CORT secretion gradually increased as the supplementation period elapsed in female rats, while changes were minimal in males. Coping strategies in the FST differed between sexes, particularly in FO-fed rats, where females and males, respectively, favoured floating and tail support to minimise energy consumption and maintain immobility. In the SIT, FO/EE promoted sociability in females, while a CSO diet favoured social recognition in males. Reduced CA3 GR-ir expression was found in FO/RC and CSO/EE rat groups, supporting stress resilience and memory consolidation. Our findings support environment and dietary conditions to exert a sex-specific impact on biobehavioural responses.

## 1. Introduction

Among essential dietary fatty acids, fish-derived omega-3 (*n*-3) polyunsaturated fatty acids (PUFAs) are required for optimal mammalian nervous system development [1,2,3,4], playing a critical role in cell membrane and synapse formation and ensuring membrane fluidity during brain maturation [1,2,3,4,5]. Over the last century, a shift towards grain-based diets has promoted increased consumption of omega-6 (*n*-6) PUFA and both saturated and trans-fat content at the expense of *n*-3 PUFA intake [1,3]. Insufficient *n*-3 dietary intake has resulted in omega-6/omega-3 consumption ratios approaching 20:1 against a recommended 4:1 [3,6]. Combined with a sedentary lifestyle, such diets pose health risks, reflected in an increased likelihood of developing cardiovascular disease, cognitive problems, and mental health disorders [1,2,3,4,5,7]. Diets rich in *n*-6 and/or deficient in *n*-3 PUFAs are associated with heightened hypothalamic–pituitary–adrenal (HPA) axis activation, glucocorticoid secretion [8,9], and depression/anxiety-related symptoms [10,11,12]. In contrast, *n*-3 supplementation has been associated with attenuation of depressive symptoms in young adults [13]. Notably, Ginty and Conklin (2015) have reported that 67% of depressed individuals no longer meet diagnostic criteria following short-term 21-day *n*-3 supplementation. Of interest, females show increased benefits from *n*-3 intake, in part related to oestrogens facilitating *n*-3 conversion into docosahexaenoic acid (DHA), which promotes brain development [14]. In turn, testosterone is known to reduce conversion rates in males [15]. Elevated dietary *n*-3 consumption in children aged 6–16 years revealed effects on cognitive performance to be twice as important in girls compared to boys [16]. While some benefits of fish oil intake during adolescence have been uncovered, additional studies are required to clarify its effects on behavioural responses and stress reactivity when consumed during this critical period.

Akin to diet, several studies have demonstrated positive behavioural changes resulting from exposure to environmental enrichment (EE), defined by the provision of heightened social and environmental stimulation through renewed external stimuli, increased physical activity, social interactions, and living space [17,18]. Studies have shown EE exposure to enhance social development in rodents, by increasing exploration and play [19], as well as to attenuate anxiety-like and depressive-like behaviour [20,21,22,23,24]. Veena et al. (2009) further supported a minimal 10-day EE exposure period to suffice in attenuating depressive-like symptoms related to chronic stress exposure in adult rodents [24], while Belz et al. (2003) showed EE to attenuate basal adrenocorticotropic hormone (ACTH) and corticosterone (CORT) secretion in male and female rats [25]. EE also promotes secretion of several neurotrophic factors [i.e., brain-derived- (BDNF), nerve- (NGF), and insulin-like growth factors (IGF) and neurotrophin-3 (NT-3)] [26] shown to impact mood regulation [27,28]. Finally, EE is associated with hippocampal neurogenesis [22,23] and improved cognitive flexibility and spatial memory performance [29].

The adolescent period, marked by increased plasticity within the frontal cortex and interconnected mesolimbic circuitries, represents a critical, time-sensitive window to evaluate the effects of diet and environment [30,31,32]. Notably, sex-dependant changes in stress reactivity related to gonadal hormone secretion [33,34] render adolescence and early adulthood distinctly vulnerable to the development of anxiety disorders and depression [14,35], with adolescent girls being twice as likely than boys to develop depression [36]. Furthermore, exposure to stressors at this period is associated with sustained elevations in glucocorticoid secretion shown alter various intracellular processes and white matter integrity in rodents’ hippocampi [37,38].

To date, published research has focused on perinatal or adulthood exposure, with a particular void being notable in social behaviour research in the context of adolescent intake, especially in females. The ability of an *n*-3 PUFA-supplemented diet and EE to regulate common endocrine and plasticity makers warrants evaluation of possible synergistic actions susceptible to affecting adolescent brain maturation and adulthood biobehavioural responses. This study therefore aims to determine sex-specific effects of single and combined exposure to dietary omega-3 supplementation and enriched environments during adolescence on (1) immediate CORT secretion and delayed regulation of glucocorticoid receptors in the CA1 and CA3 hippocampus layers, (2) coping behaviour in the forced swim test (FST), and (3) socioemotional responses as assessed in the Open Field Test, elevated-plus maze, social interaction test, and Y-maze passive avoidance test 24 h following repeated FST exposure. It is predicted that females and males fed with FO would demonstrate attenuated anxiety and heightened sociability. Moreover, while mitigated effects of EE housing have been reported in females, we expect a synergic, beneficial impact in regard to EE and FO supplementation in male rats.

## 2. Materials and Methods

### 2.1. Animals

**Figure 1:**
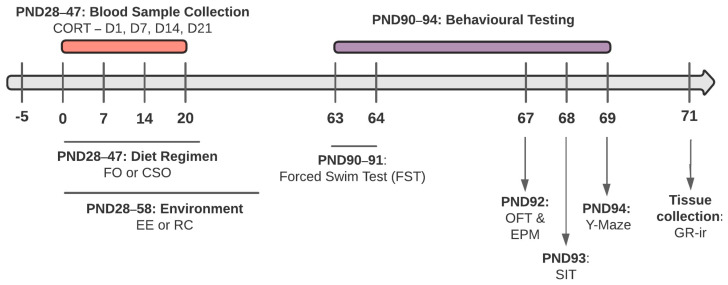
Timeline of the experiment. Wistar rats (male and female, *N* = 64) arrived at the facility at PND23. Dietary supplementation [FO or CSO] was provided daily from PND28 to 47, and rats were exposed to EE or RC from PND28 to 59. Four conditions were tested: CSO/RC, CSO/EE, FO/RC, and FO/EE. Following the FST (PND90–91), rats were exposed to the OFT, EPM, SIT and Y-Maze (PND92–94). Brain tissue was collected on PND95. *FO: menhaden fish oil; CSO: control soybean oil; EE: environmental enrichment; RC: regular cage; CORT: corticosterone; FST: forced swim test; OFT: open field test; EPM: elevated-plus maze; SIT: social interaction test; GR-ir: glucocorticoid receptors*.

Female (F) and male (M) Wistar rats (n = 32/sex) were obtained from Charles Rivers Laboratories (Rochefort, Québec, QC, Canada) and arrived at the facility on postnatal day (PND) 23. Upon arrival, rats were pair-housed with same-sex cage mates and were handled daily for 2–3 min for habituation prior to the start of the experiment. All rats received ad libitum access to regular rat chow and water and were kept on a 12 h light/dark cycle (lights on at 7 a.m.) in a temperature-controlled environment (21–23 °C) with a 40–60% relative humidity. All procedures were carried out in accordance with the Canadian Council on Animal Care (CCAC) and approved by the University of Ottawa Animal Care Committee (PY4245, renewal April 2024). Experimentations complied with ARRIVE guidelines and the National Institutes of Health Guide for the Care and Use of Laboratory Animals (see Figure 1 for timeline).

### 2.2. Dietary Supplementation and Environmental Conditions

On PND28, male and female rats were randomly assigned to one of four experimental conditions based on dietary supplementation and housing. Supplements consisted of omega-3-rich menhaden fish oil (FO; 0.3 mL/100 g body weight; F8020-1L; Sigma–Aldrich, Oakville, ON, Canada) or control soybean oil (CSO; S7381-1L; Sigma–Aldrich Canada), which were provided daily from PND28 to 47 using oral gavage, a delivery method shown to be less stressful in adolescent rats than time-restricted feeding [39]. The FO- and CSO-supplemented groups were further divided into two housing conditions: EE or regular cage (RC). For a 31-day period, EE rats were group-housed (4–5 rats) in a large cage (LWH: 44 cm × 23 cm × 20 cm) with access to acrylic tubes, paper for nesting, toys, a running wheel, and chains (for similar protocols, see [40,41]). The environment was slightly modified every 2–3 days by changing available toys/elements to maintain the rats’ interest. Following juvenile EE exposure, rats were transferred to smaller cages (LWH: 21 cm × 42 cm × 20 cm) equipped with an acrylic tube and paper for building nests without being separated from their cage mates. RC housing involved pair-housing with access to one acrylic tube. To minimise confounds related to increased exercise in EE-housed rats, RC rats were placed in an empty water maze pool (DH: 86 cm × 15 cm) for 15 min sessions three times a week. All other conditions related to housing (water and food intake) were identical between groups. In total, the four experimental conditions tested in male and female rats are identified as follows: FO/EE, FO/RC, CSO/EE, and CSO/RC.

### 2.3. Blood Sampling

From PND28, a two-drop blood sample was collected weekly between 7 and 9 a.m. over a 4-week period using the tail vein nick procedure, as previously described [42]. Blood collection took under 3 min to prevent variations in CORT secretion related to sampling stress. The two blood drops were collected on Whatman paper (Sigma–Aldrich Canada) and samples were left to dry for 24 h before being stored at −80 °C until CORT secretion analysis.

### 2.4. Estrus Cycle Assessment

Daily monitoring of females’ estrus cycle was initiated one week prior to behavioural testing through collection of vaginal samples. Briefly, a sterile pipette containing a small volume of distilled water (0.2–0.25 mL) was placed in the vaginal canal opening (<1 cm) and the liquid was gently expelled. This procedure was repeated 4–5 times to collect enough cells per sample. Fluid was transferred to a glass slide and left to dry at room temperature. Vaginal cytology was determined immediately following collection using a light microscope and the McLean et al. (2012) and Goldman et al. (2007) stage assessment guidelines [43,44].

### 2.5. Adulthood Behavioural Testing

#### 2.5.1. Repeated Forced Swim Test (FST) Exposure

As adults (PND90–91), rats were exposed to repeated FST stress. On the first day, rats were placed in a transparent plastic cylinder (DH: 28 cm × 54 cm) filled with lukewarm (20–22 °C) water for a 15 min period (as described by Lemos et al., 2012). Black cardboard was placed between each cylinder to prevent rats from seeing each other during testing, and a white curtain separated the rat testing area from the researcher’s recording station. Four to six rodents underwent testing at the same time, ensuring that female and male rats were tested in different sessions. Twenty-four hours later, rats were exposed to the same cylinder for four, six min swim sessions with 15 to 20 min between test intervals. The swim sessions acted as a repeated stressor [45]. Swim cylinders were emptied and cleaned with 70% ethanol between trials. After removal from the cylinder, rats were dried with a towel and placed back in clean cages on a heating pad for 30 min. Testing was recorded using an analogue camera (WV-CP284, Panasonic^®^, Mississauga, ON, Canada) and coded using ODlog^TM^ (Macropod 2.0 Software). Behavioural analyses were limited to the first session of the second day, as per the original FST protocol [46]. Time spent climbing, swimming, and immobile was analysed. In this context, the FST has been used to infer adaptive stress-coping strategies linked to energy conservation [47,48]. The experimenters coding the recorded behavioural responses were blind to the group conditions for all tests.

#### 2.5.2. Open Field Test (OFT)

Twenty-four hours following exposure to the FST, rats were moved to a room adjacent to the vivarium from 7 a.m. on test days to acclimate for 30 min before testing. Temperature and humidity conditions were equivalent to those of the vivarium with ~400 lux illuminations. The OFT consisted of an open square area surrounded by walls (LWH: 75 cm × 75 cm × 30 cm) made of grey opaque plexiglass and with flooring being a removable grey mat divided into 36 equally sized squares: 20 limiting the peripheral region and 16 limiting the centre zone. Rats were initially placed in one corner and allowed to move freely for 10 min. Locomotor activity and anxiety-like behaviour were assessed via latency to enter the anxiogenic centre zone (s) as well as time spent (s) and frequency of entries in centre and periphery [49]. A white curtain surrounded the behavioural testing area, preventing rats’ visual access to the researcher and to remote visual cues. Testing apparatus was cleaned with 70% ethanol between each session. Sessions were recorded using a ceiling-mounted camera (WV-CP284; Panasonic^®^, Canada) and coded using ODlog^TM^ (Macropod Software, Abbotsford, Australia). A behavioural testing sequence is depicted in the experimental timeline (see Figure 1).

#### 2.5.3. Elevated Plus Maze (EPM)

Three to four hours after the OFT, rats were tested in the EPM for 5 min to assess anxiety-like behaviour [49]. The cross-shaped apparatus was made of 2 open and 2 closed arms, each measuring 50 cm × 10 cm, attached to a central, square platform. Closed arms were surrounded by 40 cm high protective black walls, while open arms only had a 5 mm plexiglass border, exposing the rat to an open area. Rats were initially placed in the centre zone of the maze, facing an open arm. Using video camera recordings and ODlog, time spent (s) and frequency of entries in open and closed arms were determined. Risk assessment behaviours were scored when a rat dipped its head over the open arm (tip of nose up to shoulder), with its full body remaining in the closed arm or centre zone. Duration (s) and frequency of risk assessments were recorded.

#### 2.5.4. Social Interaction Test (SIT)

On PND93, the three-chamber SIT/social preference test (SP) was used to assess rodents’ social affiliation/motivation and social memory/preference for social novelty [50]. The apparatus consisted of a modified open field arena (LWH: 75 cm × 75 cm × 30 cm; standing on a Table 90 cm above the floor) with removable clear plexiglass partitions dividing the arena into three chambers of equal size. An overhead camera recorded each behaviour session. White curtains separated the experimenter’s recording area. As per Kaidanovich-Beilin et al. (2011), each rat was initially placed in the middle chamber and left to habituate to the apparatus for a 5 min period, allowing exploration of the two outer chambers, each containing identical empty wire containment cups [51]. A few minutes following habituation, the rat was placed in the middle chamber of the SIT, with a stranger rat (S1) now placed inside one of the wire cups. During the first 10 min session (assessing sociability), the rat was free to engage in direct or indirect interactions with S1 in one side chamber or spend time exploring the empty cup (EC) in the second side chamber or the middle area. For the second 10 min session (assessing social preference), the rat was returned to the middle chamber and could now freely engage in direct and indirect interactions with a novel conspecific (S2; stranger) placed under the previously empty wire cup or revisit S1. Session 2 was completed immediately following habituation and session 1. Side chamber localisation of stranger rats was counterbalanced between animals. Measures included time spent in direct contact (i.e., with the rodent’s muzzle directly touching or/and sniffing) with the conspecific or the EC in session 1, with S1 (now-familiar rat) or stranger S2 in session 2, and the time spent performing indirect behaviours in the chambers (i.e., walking, grooming, or being immobilised) while no observable direct contact with conspecifics or EC were performed [51]. The exploration ratio in the SIT was calculated as [T_S1_/(T_S1_ + T_EC_)]; thus, direct or indirect interactions with S1 were deemed elevated relative to EC when an exploration ratio > 0.5, while an exploration ratio < 0.5 indicated reduced interactions with S1 relative to the EC. In session (2), the exploration ratio was defined as [T_S2_/(T_S2_ + T_S1_)], and a ratio of >0.5 indicated an increased preference for S2 relative to S1 (i.e., social memory and predilection for novelty).

#### 2.5.5. Y-Maze Passive Avoidance Test (Y-Maze)

The Y-Maze was used to assess fear-conditioned memory [52] and consisted of a Y-shaped, 3-arm plexiglass maze (35.5 cm × 15 cm × 30 cm). One arm was used as a starting point (start arm) while the two others were possible aversive arms. Different visual cues were placed at the end of the two arms to facilitate spatial memory consolidation. Each animal was initially placed facing a wall in the start arm and left to roam freely until it entered one of the two others. Once the rat entered an arm, the exit was blocked with an opaque plexiglass sliding door and the rat received 4 puffs of air jets (dispensed every 15 s over 1 min), rendering this arm “aversive”. The rat was then removed from the arm and left to rest in its home cage for 30 min, after which it was placed back in the start arm. The initially unselected arm was now blocked by a grey plexiglass door, making the aversive arm the only available choice. If the rat failed to re-enter the aversive arm within 5 min, it was removed from the test and placed back in its home cage. Latency to enter the aversive arm (s) was logged. Time spent making risk assessments (i.e., the rat’s head—nose to shoulder—peeking into the aversive arm) was noted. Wire grates placed on top of the three arms prevented escape from the maze during testing.

### 2.6. Post-Mortem Brain Tissue Collection

On PND95, rats were lightly anesthetised using 3% isoflurane dissolved in oxygen prior to rapid decapitation. Fresh brain tissue was extracted, quickly frozen on dry ice, and stored at −80 °C prior to cryostat slicing (Leica CM1900, Leica Microsystems, Wetzlar, Germany) to collect 14 μm-thick coronal brain sections mounted on glass slides (Fisherbrand Superfrost^®^ Plus Microscope Slides, Waltham, MA, USA). Slides were stored at −80 °C until immunohistochemistry was performed. Sections were collected using coordinates from the Paxinos and Watson (2006) atlas for identification of CA1 and CA3 hippocampus subfields (−2.80 to −4.16 mm from Bregma) [53].

### 2.7. Blood Corticosterone Assessment Using Enzyme-Linked Immunosorbent Assay

An enzyme-linked immunosorbent assay (ELISA; Corticosterone EIA Kit; ADI-900-097, Enzo Life Sciences, Farmingdale, NY, USA) was used for blood CORT level determination. Briefly, 3.0 mm diameter circles were micro-punched from the Whatman collection paper using a Gem Hole puncher (McGill Inc., Fayetteville, NC, USA). Samples were placed in glass tubes filled with 200 µL of Dulbecco’s phosphate-buffered saline (DPBS; Sigma–Aldrich, St. Louis, MO, USA) containing 0.1% gelatin (Avantor Performance Materials, Phillipsburg, NJ, USA). Tubes were shaken at 90 rpm for one hour at 24 °C and refrigerated for 48 h at 4 °C. Using volumes of the buffered solution containing the dissolved blood samples, an ELISA procedure was conducted to determine CORT levels in duplicates, as previously described [54]. Following sample distribution into the wells of the ELISA plates, the primary and secondary antibodies were added and shaken at 500 rpm for 2 h at room temperature (RT) on a plate shaker. Then, plates were rinsed three times and samples were incubated in pNpp at RT for 1 h before a stop solution was added and the plates were read using a Powerwave XS2 Microplate Spectrophotometer (BioTek, Winooski, VT, USA). The assay detection range was 32–20,000 pg/mL and intra- and inter-assay consistency was confirmed.

### 2.8. Immunohistochemical Detection of Glucocorticoid Receptors (GR-ir)

Cryostat-sectioned brain tissue was pre-soaked in a paraformaldehyde fixing solution (4% PFA, 0.2% picric acid) for 15 min at RT and subsequently rinsed with 0.01 M phosphate-buffered saline (PBS; pH = 7.4; 3 × 5 min) before incubation in blocking solution (5% donkey serum; 0.2% Triton-X-100; PBS) for 30 min at RT. Tissues were rinsed prior to overnight incubation at 4 °C with the polyclonal rabbit anti-GR primary antibody (1:500; ab3578; Abcam, Toronto, ON, Canada) mixed in blocking solution. The following day, brain sections were washed in PBS (3 × 5 min) and incubated with Alexa 594-conjugated donkey anti-rabbit secondary antibody (1:1000; A-21207; Invitrogen Canada Inc., Burlington, ON, Canada) at RT in the dark for 1 h. Sections were rinsed with PBS (3 × 5 min) and immersed in Hoechst adenine–thymine-binding dye (1:20,000; Hoechst 33342, Invitrogen Canada Inc.) for 5 min at RT. Following a last series of rinses, an anti-fade medium containing 0.1% *p*-phenylenediamine in phosphate-buffered glycerol was applied, and slides were cover-slipped and sealed with nail polish. All slides were kept at −80 °C until analysis. Special controls were run to test for antibody specificity, i.e., incubation of tissue slices in PBS-Triton-Serum without the primary antibody prior to incubation in the secondary antibody.

### 2.9. Quantification of Immunoreactivity

Photomicrographs of fluorescence immunolabelling were obtained using an Olympus DX51 microscope (Center Valley, PA, USA) and the ProgRes Capture Pro 2.7.6 software under a 20× objective lens (eyepiece 10×; numerical aperture 0.75). GR-ir was manually scored by two blinded examiners and was defined as clearly visible by a circular Alexa 594 fluorescence superimposed on Hoechst dye. Four anatomically matched pictures of both hemispheres were assessed to produce an average immunoreactive score for the two brain regions of interest. The final sample sizes for females and males were as follows: FO/EE (F = 8; M = 7), FO/RC (F = 8; M = 3), CSO/EE (F = 7; M = 5), CSO/RC (F = 4; M = 7).

### 2.10. Statistical Analyses

All statistical analyses were performed using IBM© SPSS Statistics 27 (IBM, New York, NY, USA). Datasets were first screened for outliers and extreme datapoints by use of stem-and-leaf plots. If identified, such datapoints were replaced with their respective group’s now most extreme datapoint plus or minus one for high and low datapoints, respectively. Normality was then verified with skewness and kurtosis, while homogeneity of variance was confirmed using Levene’s test. Statistical corrections (square root, log10 or inverse) were performed when appropriate. Sphericity was determined using Mauchly’s test, and a Greenhouse–Geisser correction was applied when sphericity assumption was violated. Three-way analyses of variance (ANOVA) with between-factors sex (female vs. male), supplementation (fish oil vs. control soybean), and environment (enriched vs. regular) served to analyse behavioural and immunohistochemical data. CORT data were analysed using a three-way repeated ANOVA, the between factors being sex, supplementation, and environment, with the collection times as the within factor (1-, 7-, 14-, and 21 days post-supplementation). Significance was set at α < 0.05 and Bonferroni correction was applied in pairwise comparisons. Data are presented as mean ± standard error of the mean (SEM).

## 3. Results

### 3.1. Behavioural Responses in the FST

#### 3.1.1. Time Spent Climbing

**Figure 2:**
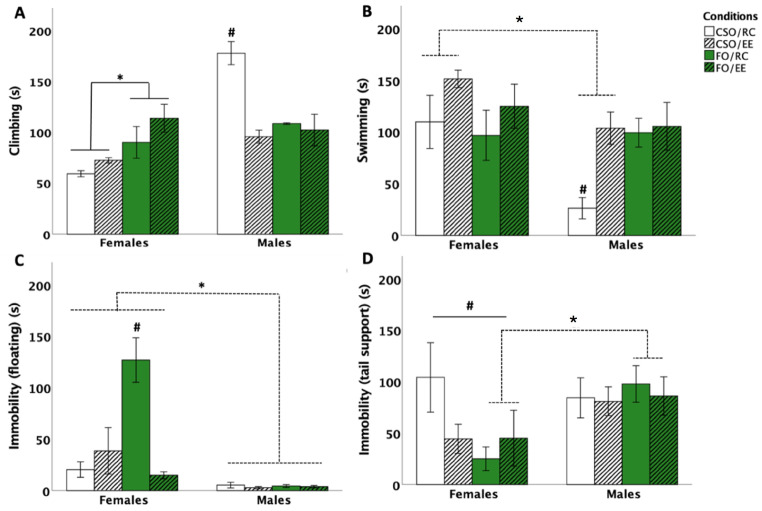
Effect of supplementation, sex, and environment in the forced swim test (FST) on time spent climbing (**A**), swimming (**B**), immobility by floating (**C**), and immobility by tail support (**D**). Increased climbing was observed for CSO/RC males compared to all groups (*p* < 0.001; #). FO Females climbed more than CSO regardless of the environment (*p* < 0.001; *; (**A**)). Swimming was enhanced in CSO-fed females compared to male counterparts in both RC and EE (*p* < 0.001; *). Male CSO/RC rats show reduced swim compared to all groups (*p* < 0.01, #; (**B**)). Immobility was increased in FO/RC females compared to all groups (*p* < 0.001; #; (**C**)). Male rats fed FO used tail support to maintain immobility (*p* = 0.001; *), while female counterparts preferred floating (*p* < 0.001; #; (**D**)). Data are presented as mean ± S.E.M. * and # indicate significant differences between groups at *p* < 0.05. * indicates significant impact of supplementation only (*p* < 0.05). *FO: menhaden fish oil; CSO: control soybean oil; EE: enriched environment; RC: regular cage*.

Three-way ANOVA revealed a main effect of sex [*F* (1,56) = 25.913, *p* < 0.001, η^2^_p_ = 0.316] and interactions between sex × environment [*F* (1,56) = 18.403, *p* < 0.001, η^2^_p_ = 0.247], sex × supplementation [*F* (1,56) = 21.301, *p* < 0.001, η^2^_p_ = 0.276], supplementation × environment [*F* (1,56) = 8.705, *p* = 0.005, η^2^_p_ = 0.826], and sex × supplementation × environment [*F* (1,56) = 4.981, *p* = 0.030, η^2^_p_ = 0.592]. The main effect of sex was related to reduced climbing in females compared to males (*p* < 0.001). Post hoc comparisons further showed climbing to be elevated in RC-housed CSO-fed rats compared to the other groups (*p* < 0.001). In addition, CSO/RC males spent the longest time climbing (three times that of CSO/RC females), a response prevented by EE (*p* < 0.001). In females, FO significantly increased climbing compared to CSO supplementation (*p* < 0.001), with the effect being observed in both RC (*p* = 0.004) and EE (*p* = 0.005) conditions. Our findings also supported CSO/EE rats to show reduced climbing compared to FO-fed counterparts (*p* = 0.022) (see Figure 2A).

#### 3.1.2. Time Spent Swimming

Three-way ANOVA revealed the main effects of sex [*F* (1,56) = 7.723, *p* = 0.007, η^2^_p_ = 0.121], supplementation [*F* (1,56) = 8.262, *p* = 0.006, η^2^_p_ = 0.129], and the interaction between sex × supplementation [*F* (1,56) = 4.6, *p* = 0.036, η^2^_p_ = 0.076] on the time spent swimming. Pairwise comparisons indicated that being a male significantly reduced swim time (*p =* 0.007). Additional post hoc analyses revealed CSO supplementation to be associated with increased swim time in females compared to that of male counterparts (*p* < 0.001). CSO-fed male rats exposed to RC spent less time swimming compared to all other male and female groups (*p* < 0.01 for all comparisons). This effect is attributable to CSO/RC male rats favouring climbing over swimming. EE exposure acted to normalise swimming behaviour in CSO-fed male rats (see Figure 2B).

#### 3.1.3. Time Spent Immobile: Floating and Tail Support

In this study, rats were not exposed to chronic or repeated stress exposure and were thus unlikely to develop depressive-like symptoms. As such, the FST was used to assess coping and energy conservation. To characterise sex-specific coping strategies, tail support was enabled as an alternate strategy to passive floating [as described by Abelaira et al. (2013)]. Although no changes in overall time spent immobile were observed between the groups, separate coping strategies were found to be influenced by sex, diet, and housing conditions. In the case of floating, three-way ANOVA revealed the main effects of sex [*F* (1,56) = 35.877, *p* < 0.001, η^2^_p_ = 0.390], supplementation [*F* (1,56) = 7.234, *p* = 0.009, η^2^_p_ = 0.114], and environment [*F* (1,56) = 9.906, *p* = 0.003, η^2^_p_ = 0.150], as well as significant interactions between sex × supplementation [*F* (1,56) = 7.230, *p* = 0.009, η^2^_p_ = 0.114], sex × environment [F (1,56) = 8.685, *p* = 0.005, η^2^_p_ = 0.134], supplementation × environment [*F* (1,56) = 17.347, *p* < 0.001, η^2^_p_ = 0.237], and sex × supplementation × environment [*F* (1,56) = 18.369, *p* < 0.001, η^2^_p_ = 0.247]. Pairwise comparisons revealed that females used floating to maintain immobility for significantly longer compared to males (*p* < 0.001). FO/EE rats floated less than the FO/RC condition (*p* < 0.001). Floating was maximal in FO-fed females and significantly reduced by EE housing (*p* < 0.001). FO-fed females also spent considerably more time floating compared to all male rats (*p* < 0.001) and CSO-fed females regardless of housing environments (*p* < 0.001). Females in the FO/RC condition spent more time floating compared to all other conditions (*p* < 0.001 for all comparisons; see Figure 2C).

For tail support, a main effect of sex [*F* (1,56) = 4.661, *p* = 0.038, η^2^_p_ = 0.075] was observed. Post hoc analyses revealed that females spent significantly less time using tail support compared to males (*p* = 0.035). Analyses also showed an interaction between sex, supplementation, and environment, attributable to FO-fed males (exposed to RC or EE) spending more time using tail support compared to similarly housed FO females (*p* = 0.001). In contrast, CSO/RC females largely used tail support to maintain immobility compared to all other female groups (*p* < 0.01 for each group comparison). EE housing significantly reduced immobility using tail support in CSO females (*p* < 0.01; see Figure 2D).

### 3.2. Open Field Test (OFT)

#### 3.2.1. Time in the Centre Zone

**Figure 3:**
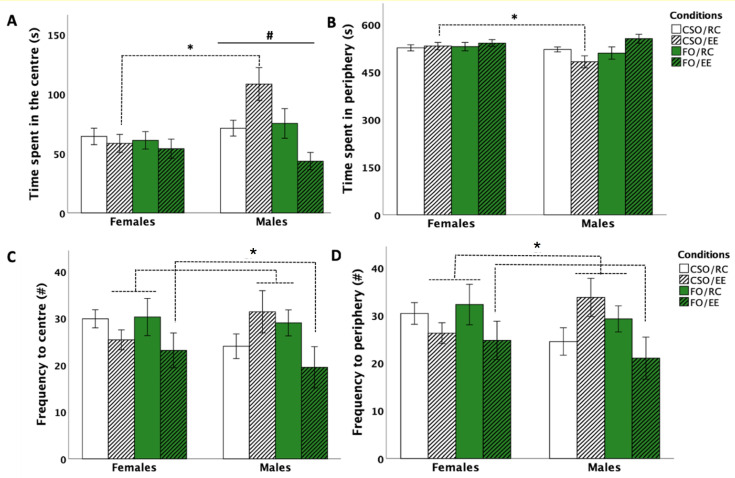
Effect of supplementation, sex, and environment in the Open Field Test (OFT) for time spent in the centre (**A**), time spent in the periphery (**B**), frequency to centre (**C**), and frequency to periphery (**D**). Male rodents spent more time in the centre zone compared to females (*p* < 0.05; #). CSO/EE males spent increased time in the centre zone (**A**), while females in the same condition spent increased time in the periphery ((**B**); *p* < 0.05; *). Rats in the FO/RC and CSO/EE group also entered more frequently the centre (**C**) and peripheral zone (**D**) compared to the FO/EE condition (*p* < 0.05; *). Data are presented as mean ± S.E.M. * and # indicate a statistically significant difference between groups (*p* < 0.05). * indicates significant impact of the condition (supplementation and housing only) with no effect of sex (*p* < 0.05). *FO: menhaden fish oil; CSO: control soybean oil; EE: enriched environment; RC: regular cage*.

Analyses were performed on log10-transformed data to correct for variance heterogeneity. Three-way ANOVA revealed the main effects of sex [*F* (1,56) = 4.486, *p* = 0.023, η^2^_p_ = 0.089], supplement [*F* (1,56) = 6.988, *p* = 0.011, η^2^_p_ = 0.111], and interactions between sex×supplement [*F* (1,56) = 4.220, *p* = 0.045, η^2^_p_ = 0.070], supplement×environment [*F* (1,56) = 7.303, *p* = 0.009, η^2^_p_ = 0.115], and sex×supplement×environment [*F* (1,56) = 6.851, *p* = 0.011, η^2^_p_ = 0.109]. Post hoc tests indicated reduced time spent in the centre zone in female rats compared to male rats (*p* = 0.023). CSO-fed rats spent increased time in the centre zone compared to FO (*p* = 0.011). The interaction between sex and supplementation was related to CSO-fed females spending reduced time in the centre zone compared to CSO-fed males (*p* = 0.003). EE potentiated centre exploration of CSO but not FO-fed rats (*p* < 0.01). Lastly, the sex × supplementation × environment interaction was related to CSO/EE males spending increased time in the centre zone compared to CSO/EE females (*p* < 0.01; see Figure 3A).

#### 3.2.2. Time in the Peripheral Zone

Analyses revealed significant interactions between environment × supplementation [*F* (1,56) = 5.319, *p* = 0.025, η^2^_p_ = 0.087] and environment × supplementation × sex [*F* (1,56) = 4.042, *p* = 0.049, η^2^_p_ = 0.067]. Post hoc analyses indicated these differences to be attributable to CSO/EE females spending more time in the periphery compared to the CSO/EE male counterparts (*p* = 0.012; see Figure 3B).

#### 3.2.3. Frequency of Entries in the Centre and Peripheral Zones

Analyses of centre entry frequencies indicated a supplementation × environment interaction [*F* (1,56) = 4.105, *p* = 0.048, η^2^_p_ = 0.068], attributable to CSO/EE (*p* = 0.043) and FO/RC (*p* = 0.018) groups entering the centre zone more frequently compared to FO/EE rats (See Figure 3C). For entries in the periphery, analyses revealed a supplementation × environment interaction [*F* (1,56) = 4.553, *p* = 0.037, η^2^_p_ = 0.075] related to rats in the CSO/EE and FO/RC conditions making more entries in the periphery compared to FO/EE rats (*p* = 0.044 and *p* = 0.027, respectively; see Figure 3D).

### 3.3. Elevated Plus Maze (EPM)

**Figure 4:**
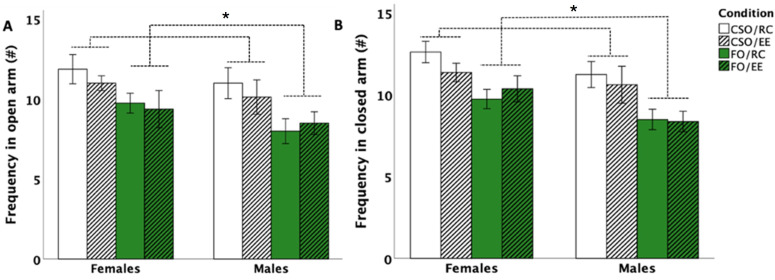
Effect of supplementation, sex, and environment in the Elevated Plus Maze (EPM) for frequency in the open arm (**A**) and closed arm (**B**). FO-supplemented rats entered the open (**A**) and closed arms (**B**) less frequently than CSO-supplemented rats (*p* < 0.001; *). Data are presented as mean ± S.E.M. * indicates a significant impact of the condition (supplementation only) without an influence of sex or housing at *p* < 0.05. *FO: menhaden fish oil; CSO: control Soybean oil; EE: enriched environment; RC: regular cage*.

EPM data indicated no significant changes in the time spent in the open or closed arms or in the number of risk assessments (all *p* > 0.05). For frequencies of arm entries, analyses indicated the main effects of supplementation for the open [*F* (1,56) = 11.721, *p* = 0.001, η^2^_p_ = 0.173] and closed [*F* (1,56) = 17.759, *p* = 0.001, η^2^_p_ = 0.241] arms. CSO-fed rodents tended to enter the open arms more frequently compared to FO-fed counterparts (*p* < 0.001; see Figure 4A,B).

### 3.4. Social Interaction Test (SIT)

#### 3.4.1. Social Interaction

**Figure 5:**
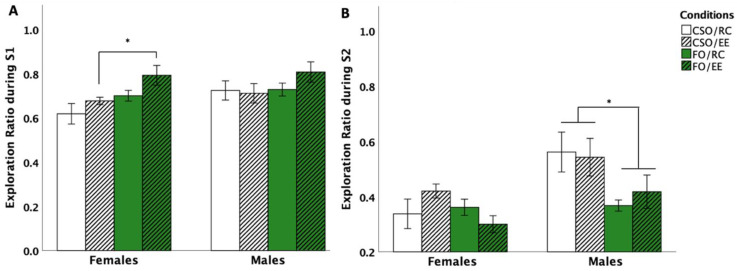
Effect of supplementation, sex, and environment in the Social Interaction Test (SIT) for exploration in session 1 (**A**) and session 2 (**B**). FO/EE females spent more time interacting with S1 than the empty cup compared to CSO/EE counterparts (*p* < 0.05; *), supporting increased sociability (**A**). FO supplementation in males reduced social recognition through reduced interaction time with S2 compared to CSO-fed counterparts (*p* < 0.05; *), notwithstanding housing conditions (**B**). Data are presented as mean ± S.E.M. * indicates a statistically significant difference between groups at *p* < 0.05. *FO: menhaden fish oil; CSO: control soybean oil; EE: enriched environment; RC: regular cage*.

SIT data were analysed using chamber exploration time ratios. Changes in sociability involved a ratio calculated from time spent interacting with S1 versus total exploration time (S1 and empty cup exploration) [i.e., T_S1_/(T_S1_ + T_EC_)]. Statistical analyses revealed the main effects of supplementation [*F* (1,56) = 7.546, *p* = 0.008, η^2^_p_ = 0.129] and environment [*F* (1,56) = 4.005, *p* = 0.050, η^2^_p_ = 0.090]. Overall, post hoc comparisons showed the FO diet to promote exploration of S1 compared to EC (*p* = 0.05). No main effect of sex emerged. However, due to an a priori interest in sex differences [55] in the context of this study, planned comparisons were performed, which showed increased time exploring S1 versus EC in FO-fed females compared to CSO-fed counterparts. In males, no significant changes in sociability were noted (see Figure 5A).

#### 3.4.2. Social Preference

Changes in social recognition were assessed using a ratio between time spent interacting with S2 versus total exploration time (S1 and S2) [i.e., T_S1_/(T_S1_ + T_EC_)]. Analyses revealed the main effects of supplementation [*F* (1,56) = 8.98, *p* = 0.004, η^2^_p_ = 0.138], sex [*F* (1,56) = 11.52, *p* = 0.001, η^2^_p_ = 0.171], supplement×sex [*F* (1,56) = 8.674, *p* = 0.005, η^2^_p_ = 0.171], and environment×supplement×sex [*F* (1,56) = 8.578, *p* = 0.005, η^2^_p_ = 0.151] interactions. Post hoc comparisons revealed CSO to promote social recognition in male rats through increased S2 exploration compared to FO counterparts (*p* = 0.004), independent of housing conditions. CSO-fed males also interacted more with S2 compared to CSO-fed females (*p* = 0.001) (see Figure 5B).

### 3.5. Y-Maze Avoidance Test

#### 3.5.1. Latency to Aversive Arm Entry

**Figure 6:**
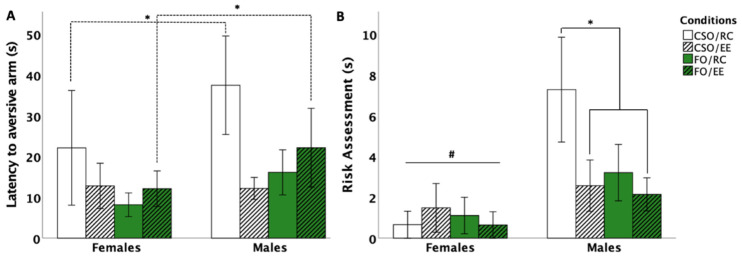
Effect of supplementation, sex, and environment for latency to arm re-entry (**A**) and Risk assessment behaviour (**B**) in the Y-maze passive avoidance test. In general, males CSO/RC and FO/EE took more time to re-enter the aversive arm compared to females in the same condition (*p* < 0.05; *; (**A**)). In males, CSO/EE and FO/EE showed reduced assessments compared to the CSO/RC condition (*p* < 0.05; *). Females underwent fewer risk assessments compared to males (*p* < 0.05; #; (**B**)). Data are presented as mean ± S.E.M. * and # indicate a statistically significant difference between groups at *p* < 0.05. *FO: menhaden fish oil; CSO: control soybean oil; EE: enriched environment; RC: regular cage*.

Analyses revealed a main effect of sex [*F* (1,56) = 7.385, *p* = 0.005, η^2^_p_ = 0.132] as well as sex×environment [*F* (1,56) = 4.719, *p* = 0.034, η^2^_p_ = 0.078] and environment × supplementation [*F* (1,56) = 5.889, *p* = 0.018, η^2^_p_ = 0.095] interactions. Post hoc comparisons indicated reduced latency for aversive arm entry in females compared to males (*p* = 0.005). Interestingly, reduced latencies (i.e., fear conditioning) in FO-fed females compared to males were observed in EE-housed animals (*p* = 0.028), while a similar sex difference was observed in RC-housed, CSO-fed females compared to males *(p* = 0.008). In CSO-fed males, but not females, EE housing significantly reduced latencies to re-enter the aversive arm compared to RC housing (*p* = 0.033; see Figure 6A).

#### 3.5.2. Risk Assessment Behaviour

Analyses indicated a main effect of sex (*F* (1,56) = 9.092, *p* = 0.004, η^2^_p_ = 0.128) and interactions between environment×supplement (*F* (1,56) = 3.7, *p* = 0.016, η^2^_p_ = 0.156) and sex×environment (*F* (1,56) = 3.378, *p* = 0.018, η^2^_p_ = 0.158). Post hoc comparisons indicated reduced risk assessments in females compared to males (*p* = 0.03 for RC- and *p* = 0.04 for EE-housed rats). Within male groups, CSO/RC rats made significantly more risk assessments compared to CSO/EE and FO/EE conditions (*p* = 0.014 and *p* = 0.033, respectively; see Figure 6B).

### 3.6. Corticosterone Level

**Figure 7:**
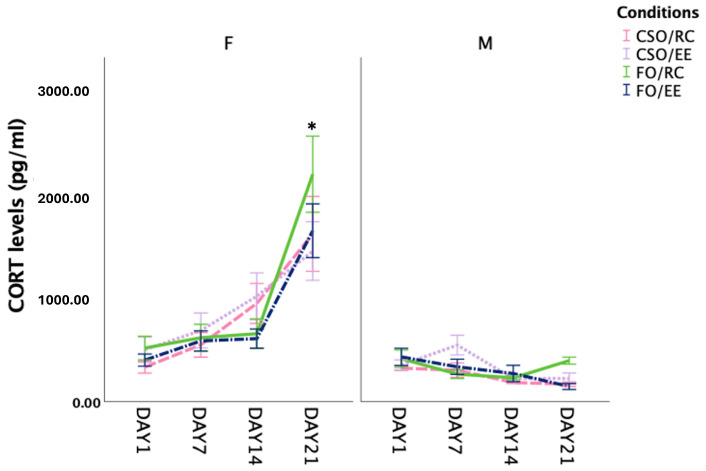
Corticosterone levels (pg/mL) assessed on experimental DAY1, 7, 14, 21. Statistical increases in CORT were observed for all females compared to males on DAY21 (*p* < 0.001) as well as between females from DAY1, 7, and 14 (*p* < 0.001 for each day). Data are presented as mean ± S.E.M. * indicates a statistically significant difference between groups at *p* < 0.05. *FO: menhaden fish oil; CSO: control soybean oil; EE: enriched environment; RC: regular cage*.

A repeated measure ANOVA revealed a sex×time [*F* (1,53) = 10.781, *p* = 0.035, η^2^_p_ = 0.655] interaction, attributable to higher CORT levels in females compared to male rats. Pairwise comparisons revealed CORT levels to be significantly elevated on DAY21 compared to DAY1 (*p* < 0.001), DAY7 (*p* < 0.001), and DAY14 (*p* < 0.001). No significant effects of dietary supplementations or housing conditions were found (see Figure 7).

### 3.7. Glucocorticoid Receptor Immunoreactivity

**Figure 8:**
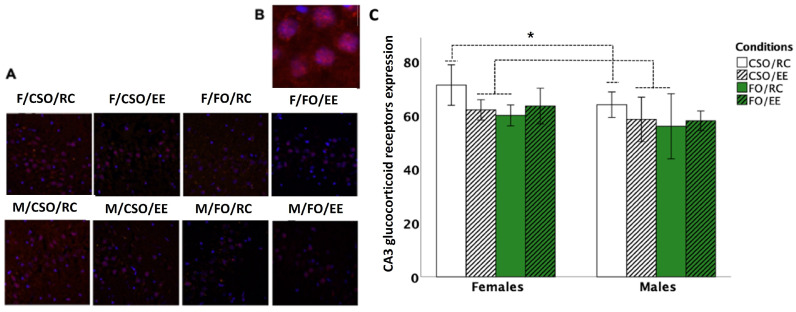
GR-ir at the CA3 region of the hippocampus. Figure shows representative photomicrographs of GR-ir in the CA3 for each experimental condition (**A**) as well as specificity of GR antibody through superposition on Hoechst adenine–thymine-binding dye (**B**). Reduced GR-ir was observed in CSO/EE and FO/RC groups compared to CSO/RC rats (*p* = 0.019 and *p* = 0.005, respectively; (**C**)). Data are presented as mean ± S.E.M. * indicates effects of supplementation and housing without effects of sex (*p* < 0.05). *FO: menhaden fish oil; CSO: control soybean oil; EE: enriched environment; RC: regular cage; GR: glucocorticoid receptors*.

Three-way ANOVA on GR-ir density revealed no group differences at the CA1 (*p* > 0.05). At the CA3, analyses revealed the main effect of sex [*F* (1,42) = 5.577, *p* = 0.023, η^2^_p_ = 0.117], supplementation [*F* (1,42) = 4.574, *p* = 0.038, η^2^_p_ = 0.098], and a supplementation × environment [*F* (1,42) = 5.519, *p* = 0.024, η^2^_p_ = 0.116] interaction. Pairwise comparisons indicated reduced GR-ir expression in FO- compared CSO-fed rats (*p* = 0.038). The effect of sex is attributable to reduced GR-ir in males compared to female counterparts (*p* = 0.023). Finally, CSO/RC groups showed elevated CA3 GR-ir compared to CSO/EE (*p* = 0.019) and FO-RC (*p* = 0.005) groups (see Figure 8).

## 4. Discussion

This study aimed to assess sex-specific effects of combined *n*-3 dietary supplementation and environmental enrichment exposure during adolescence on delayed adulthood coping mechanisms, stress response, and sociability. Our findings support the differential effects of treatments administered individually, or in combination, on studied parameters across both sexes.

### 4.1. Sexually Dimorphic Influence of FO Supplementation and Housing on Coping Response

On PND90–91, all rats were exposed to the FST. Although often used to infer depressive-like behaviour in rodents exposed to various experimental paradigms and stressors [46], several studies highlight the importance of immobility in the FST as an adaptative strategy promoting energy conservation [56,57]. Thus, de Kloet and Molendijk (2016) demonstrated that elevated CORT secretion and hippocampal GR activation seen upon rescuing rats from the cylinder facilitates storage of this event in memory, leading to the adoption of a preferred, mostly passive, coping style during re-exposure to the FST [48,58]. This perspective provides an interesting scope to evaluate sex-, housing-, and diet-related effects in the FST.

Our findings revealed FO supplementation in females to be associated with a distinctive increase in immobility (i.e., passive coping) compared to all other conditions. Colom-Lapetina et al. (2017) identified heightened immobility to be related to learning and memory consolidation, especially in a familiar environment. In other words, using additional resources to escape the apparatus (active coping) is deemed unnecessary, as experience indicates the swim session will end shortly [59]. In males, CSO-supplemented, RC-housed rats preferred active coping, showing a ~2–3.5-fold increase in time spent climbing compared to all other groups. Several studies have demonstrated a strong association between FO supplementation and memory consolidation, especially in females [16,60]. Although brain circuitries remain to be fully characterised, passive coping responses, such as tail-supported immobility or free floating, have been shown to depend on efficient dorsolateral striatal and limbic learning circuits and to involve hippocampal GRs as essential parts of consolidating prior FST experience [48,61]. Warden et al. (2012) have also demonstrated medial prefrontal cortex (mPFC) activation to act as an on/off switch for active versus passive behavioural selection in the FST. They highlighted that triggering a cluster of mPFC neurons that project to the brainstem’s serotonergic dorsal raphe nucleus (DRN) induced a profound, rapid, and reversible selection of the active behavioural state in the FST [62]. Future research should thus address potential sex-specific differences in coping responses resulting from the activation of such pathways.

In this study, FO-supplemented, RC-housed females showed a trend for higher CORT elevations as juveniles, potentially leading to differential priming of adulthood FST-induced GR activation. Such responses could have enabled FO/RC females to better attend to cues linked to the stressful swim exposure and, by extension, enhanced consolidation of this experience [58]. Importantly, EE housing prevented FO-induced immobility in females, supporting the synergistic actions of diet and environmental conditions. This finding is particularly interesting as it suggests that juvenile experiences exert a significant impact on modelling response patterns upon acute stress exposure. Considering that rat groups in this study were not exposed to commonly used stress paradigms that lead to depressive-like behaviour in the FST, more studies are required to better understand and interpret the singular impact of EE noted in FO-fed females.

Interestingly, male rats favoured alternative strategies to conserve energy, with a more frequent selection of tail support to maintain immobility compared to female rats, a finding especially manifested when comparing the FO/RC male and female groups. This experimental condition appears as most effective in promoting the use of passive coping strategies in both sexes, albeit using different strategies. Our results suggest that fish oil supplementation and environmental enrichment do not share common effects in regulating coping strategies upon stress exposure.

### 4.2. Juvenile FO Supplementation in Non-Deficient Rats Has a Marginal Impact on Adulthood Anxiety Levels

In the OFT, male rats showed increased time exploring the centre zone compared to female counterparts. The CSO/EE males spent increased time in the centre zone and performed increased frequencies of entries in the centre and peripheral OFT zones, which suggests increased locomotor exploration and reduced anxiety-like behaviour. Consistent with observations, Teixeira et al. found that combined exposure to CSO- supplementation and exercise in mice reduced anxiety-like behaviour, as demonstrated by greater head-dipping frequency and longer time spent in open arms, whereas exercise did not improve these parameters in lard and hydrogenated vegetable fat-fed rats or rats fed CSO alone [63]. Anxiolytic effects in CSO-exercised mice were associated with higher activity of Na^+^K^+^-ATPase in the cortex and hippocampus, favouring cell membrane fluidity. Interestingly, soybean oil supplementation has been shown to enhance serotonin or tryptophan availability [64,65,66] and to increase tryptophan and serotonin secretion in cortical tissues [67]. Such actions could contribute to combined CSO/EE attenuating some of the responses associated with anxiety in the OFT. Additional studies on the biochemical effects of juvenile FO and CSO supplementation in males and females are warranted to confirm the mechanisms involved.

Surprisingly, considering reported anxiolytic effects of *n*-3 PUFA-enriched diets [68], rat groups in this study did not differ in the time spent in the EPM open arms. However, reduced frequencies of arm entries were noted in FO-fed male and female rats compared to CSO-fed counterparts independently of housing conditions. Increases in entries were also observed in female versus male rats. Increased exploration associated with soybean supplementation has previously been noted, most likely modulated through the serotonergic and dopaminergic pathways [63,69]. For instance, Yimit and colleagues [69] reported that daily soybean supplementation increased serum dopamine levels while reducing adrenaline secretion in healthy subjects, an effect that could influence locomotion. Additionally, a study by Teixeira et al. demonstrated increased locomotion and exploratory behaviour in male rodents supplemented with soybean oil compared to those receiving hydrogenated vegetable fat or lard [63]. Therefore, CSO supplementation could have enhanced exploration through regulation of serotonergic and/or dopaminergic pathways.

Several examples of research have also associated *n*-3 PUFA deficiency with increased anxiety-like behaviours [14,35,70,71]. Our findings indicate that juvenile FO supplementation in non-deficient juvenile rats does not alter adulthood anxiety levels. However, FO alone reduced the number of EPM open and closed arm entries in both male and female rats compared to CSO-fed counterparts, possibly indicating reduced novelty-induced arousal. Consistent with this, Thesing et al. (2018) showed reduced plasma DHA levels to be accompanied by HPA axis dysregulation, leading these authors to propose DHA to regulate physiological stress and improve stress-related behaviours [72]. Consistent with this, Rice and al. (2015) reported omega-3 deficiency to be associated with elevated glucocorticoid levels and depression-like symptoms [35].

### 4.3. FO and EE Fostered Sociability in Females While CSO Promoted Social Recognition in Male Rats

In the SIT, our findings support exposure to *n*-3 PUFA supplementation and EE to exert sex- and context-specific effects on adulthood sociability and social recognition. FO fosters sociability in females through increased time exploring the stranger rat rather than the empty cage compared to CSO supplementation, with no significant impact in regard to housing. This observation is consistent with studies highlighting the key roles of *n*-3 PUFA in regulating prosocial behaviour, impulsive mood, and negative interactions in different species [73], including effects regarding reducing violent and aggressive behaviour in rodents, dogs, and humans [74,75,76]. Although not expected, the absence of EE effects is consistent with previous reports of reduced centre-chamber time but not increased interaction time with the stranger rat in adolescent EE-exposed rodents [77]. In this context, it appears that the social and physical components of enrichment may play distinctive roles in promoting sociability, as increased social enrichment itself does not alter social behaviour, while housing in larger cages equipped with physical stimuli increases social affiliation [78].

A differential impact of diet and housing conditions was noted in assessing social recognition, where soybean supplementation and regular housing acted to increase interactions with the novel congener in male, but not female, rats. A recent review by Aspesi and Choleris (2021) supports social recognition being regulated in a sex-specific manner by a host of steroids and mediated in males by direct interaction of the androgen metabolites 3α-diol and 3β-diol with oxytocin and vasopressin, affecting other downstream systems and behaviours (e.g., GABA_A_ and anxiety-related behaviours; [79,80]). In this context, Su et al. (2021) noted a 20% dietary CSO supplementation to significantly increase the luteinising hormone (LH) and testosterone levels in male mice, possibly driving observed effects in our study [81]. In addition, Choi et al. (2009) showed increased cortical tryptophan and serotonin secretion in male rats supplemented with soy protein, proposed to regulate enhanced cognitive performance associated with a 4-week exposure to the diet [67,82]. In contrast, Watanabe et al. (2010) demonstrated a significant reduction in serotonin turnover in the hypothalamic region following a PUFA-supplemented diet, related to fish oil’s hypophagic effects [83]. Characterisation of biochemical effects of adolescence-targeted supplementation in males and females is warranted to confirm the mechanisms involved.

### 4.4. Juvenile Dietary Supplementation Influenced Adulthood Fear Responses in a Sex-Dependent Manner

In the Y-Maze, FO combined with EE prevented fear conditioning in female compared to male counterparts. This is consistent with studies showing DHA supplementation and EE to promote stress resilience and adaptation to aversive environments [23,84]. CSO supplementation similarly led to sex-specific alterations marked by increased conditioned fear memory (through increased risk assessments and latency to enter the aversive arm) in male rats, an effect prevented by EE housing [23,84]. These results, along with observations of increased risk assessment in CSO/RC males compared to other male and female groups, could support better resilience to stress exposure in FO-fed and EE-exposed groups. Consistent with this, Mora-Gallegos and Fornaguera (2019) showed that enhanced social contact and EE housing at a young age reduces anxiety and fear conditioning [85]. Moreover, Barbelivien et al. (2006) reported that female exposure to EE attenuated fear conditioning responses in rodents exposed to aversive stimuli, possibly related to EE-housed female rodents being able to process contextual information faster [86].

### 4.5. Diet and Housing Exerted Distinctive Effects on Adulthood GR-ir Expression at the Hippocampal CA3 Layer

Assessed on PND95, a lasting impact of diet and housing conditions was observed in GR-ir expression in the hippocampal CA3 region, with no significant changes observed on the CA1 layer. Notably, juvenile exposure to CSO or to EE alone was associated with reduced expression at the CA3 in both sexes, which contrasts with elevated GR-ir in CSO/RC-exposed rats. Interestingly, de Quervain et al. (2009) proposed that elevated hippocampal GR expression impairs memory retrieval and working memory [87]. In this context, reduced GR-ir at the CA3 in CSO/EE and FO/RC exposed rats could have contributed to increased resilience upon adulthood exposure to the FST [48], a response not observed in CSO/RC rats. In humans, *n*-3 PUFA-rich diets have shown effects in mitigating CORT-induced apoptosis in hippocampal neurons [88]. Our findings at the CA3 in EE-treated groups, however, contrast with elevated GR expression previously associated with juvenile or adulthood EE exposure [89,90,91] or reduced GR expression in EE exposed rodents [92]. In sum, considering that EE benefits were accentuated when combined with CSO, but not FO, more studies are warranted to better understand the specific impact of diet or environmental enrichment on the functional regulation of hippocampal circuitry, as changes affecting the ventral CA3 regions may have a more decisive impact on adulthood regulation of socio-affective states. In this context, numerous studies have supported the beneficial effects of EE housing on learning and memory and stress-related emotional reactivity [93,94]. Morphologically, EE-related changes have included increased cortical thickness, synaptic and dendritic spines densities, and hippocampal neurogenesis, fostering long term potentiation in hippocampal neurons [93]. EE associated molecular changes have also been shown to impact the noradrenergic, cholinergic, and serotonergic pathways, which could also play a role in the changes observed in this study.

### 4.6. Limitations

This study examined a complex interplay between physiological and behavioural systems. Although findings from animal studies present a range of possibilities compared to those conducted in humans, issues of translatability do present a challenge. First, we noted FO supplementation to elicit a somewhat aversive response compared to CSO when fed to juvenile rats, possibly acting as a stress factor. While the gavage procedure itself is widely used to administer supplements, Brown et al. (2000) demonstrated that giving oil supplements (soybean, sesame or peanut oils) by gavage can increase CORT levels for up to 4 h following the procedure, although habituation appears to be achieved in a few days [95]. Thus, it is assumed that FO gavage worked in a similar way and that animals gradually adapted to supplementation. Using the same feeding protocol, we have demonstrated sex-specific changes in CORT secretion over a supplementation period marked by increased CORT levels measured on the last supplementation day, which reached significance only for female rats. Male rats showed no impact of gavage on CORT secretion measured over days (blood droplets were collected on supplementation days 1, 7, 14, and 21 [39]). Lastly, to focus on the cognitive benefits of EE experience and minimise benefits from exposure to physical exercise only, our study provided RC-exposed rats with intermittent exposure to a large, empty Morris water maze pool arena where they could freely explore. At present, we have found no studies that used similar measures, rendering it difficult to document the effects of these exploration sessions. We, however, believe that such a procedure could enhance translation and help best extract the contribution of environmental enrichment compared to using conventional housing [96]. Nonetheless, the resemblance of this open space to the Open Field arena could have influenced the OFT behavioural responses of RC-housed rats, although the findings do not support this factor having played a decisive role in RC exploration and/or anxiety-like responses in the OFT.

## 5. Conclusions

This study is the first to assess the combined, delayed effects of dietary omega-3 supplementation and exposure to environmental enrichment during the juvenile period on adult biobehavioural responses. The non-synergistic and sex-specific effects of dietary and environmental conditions support distinct, complex physiological mechanisms regulating assessed behaviours. Importantly, our findings support juvenile *n*-3 PUFA supplementation in females and males being associated with passive coping strategies, as well as the effects of EE exposure being sex-dependent. Additional observations indicate that CSO supplementation benefited males’ emotional response profile, which was not expected. While our findings failed to confirm that FO exposure would induce enhanced benefits in regard to social and emotional behaviour in male rodents, such a diet generated positive effects in female rodents. Finally, assessing juvenile CORT secretion over the supplementation period highlighted differential CORT secretion profiles in male and female rats as the supplementation period elapsed. Together, our findings highlight the intricate effects of dietary and environmental influence experienced at a critical developmental stage on immediate and delayed HPA activation and stress coping strategies.

## Data Availability

The original contributions presented in the study are included in the article, further inquiries can be directed to the corresponding author.
